# Metastatic breast cancer presenting as sequential cranial nerve palsy: a case report

**DOI:** 10.1186/1752-1947-8-430

**Published:** 2014-12-16

**Authors:** Indira M Madgula, Christopher M Hemmerdinger, Peter Clark

**Affiliations:** 16 Ophthalmology Department, Warrington Hospital, Lovely Lane, Warrington, WA5 1QG UK; 17 Clatterbridge Cancer Center NHS Foundation trust, Clatterbridge Hospital, Clatterbridge Road, Bebington, Wirral, Merseyside, CH63 4JY UK

**Keywords:** Breast cancer, Carcinomatous meningitis, Cranial nerve palsy, Metastases

## Abstract

**Introduction:**

Cranial nerve palsy is a common presentation in the neuro-ophthalmology clinic and investigations are directed towards the cause. Metastatic breast cancer presenting as carcinomatous meningitis leading to sequential fourth, third and sixth nerve palsy is very rare. This is the first case to be reported to the best of our knowledge.

**Case presentation:**

A 66-year-old Caucasian woman presented with vertical double vision for the previous 3 weeks. At 6-weeks follow up this had resolved. However, she presented with a new third and sixth cranial nerve palsy. Neuroimaging with contrast revealed carcinomatous meningitis.

**Conclusions:**

Metastatic cancer may manifest as cerebral metastases or carcinomatous meningitis. This is evident on neuroimaging with contrast and may be missed on unenhanced scans.

## Introduction

Carcinomatous meningitis has been reported in 5% of breast cancers and can present with headache, cranial nerve dysfunction, seizures and intracranial hypertension signals [1]. Virtually all cases occur late in the natural history of breast cancer after considerable amounts of systemic treatment. Prognosis is poor and treatment options limited [2, 3].

It results from metastatic infiltration of the leptomeninges by malignant cells leading to central nervous system dysfunction [4]. The case described here is rare because carcinomatous meningitis manifested as cranial nerve palsy in a woman without a known history of breast cancer.

## Case presentation

A 66-year-old Caucasian woman presented with vertical double vision for the previous 3 weeks. Diplopia was binocular and noticed after she banged her head against the shower screen. She was healthy and had no symptoms suggestive of giant cell arteritis (GCA). A clinical examination showed right fourth cranial nerve palsy. Her magnetic resonance imaging (MRI) scan was normal. Her erythrocyte sedimentation rate (ESR) was 77mm/hour and C-reactive protein 68mg/L. She was commenced on oral steroids while awaiting temporal artery biopsy (TAB) to rule out GCA. TAB revealed normal histology. As there was no clinical suspicion of GCA, her steroids were stopped. The medics investigated her further for raised inflammatory parameters and no cause was identified.

She then sought medical attention for a breast lump that she had noticed for the past few months. This was clinically diagnosed as breast cancer. She subsequently underwent right wide local excision of the mass and axillary clearance. Histology revealed 28mm grade 3 oestrogen receptor positive, human epidermal growth factor receptor 2 negative ductal carcinoma involving 12 of 14 axillary nodes. An oncology referral was made.

**Figure 1:**
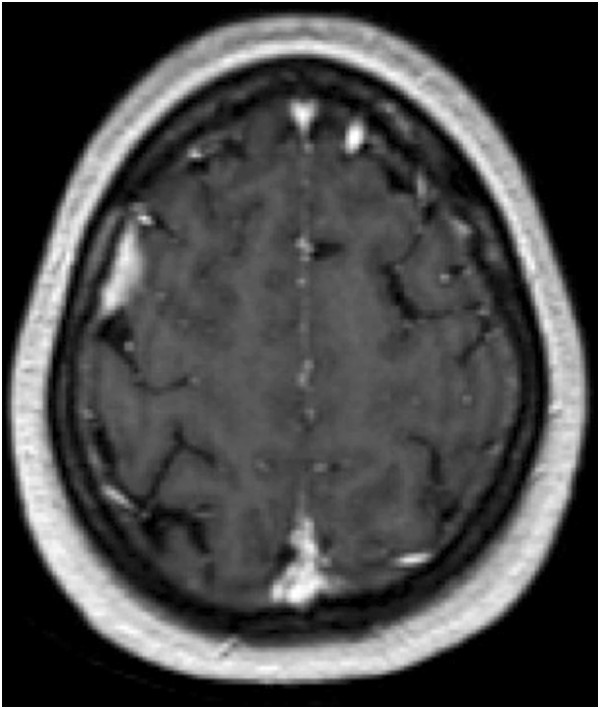
**Magnetic resonance imaging with contrast shows enhancement of the meninges in the frontal, parietal and occipital lobes consistent with carcinomatous meningitis.**

Meanwhile, she complained of a droopy right eyelid. Orthoptic examination showed pupil-sparing third nerve paresis and mild sixth nerve involvement. The fourth nerve paresis had resolved. Neuroimaging to rule out cavernous sinus pathology was requested. A MRI scan with contrast showed meningeal thickening in frontal, parietal and occipital lobes and no abnormality in the cavernous sinus territory. This was consistent with carcinomatous meningitis (Figure 1).

By the time of her oncology appointment, she had developed numbness in the ophthalmic division of her right trigeminal nerve. A bone scan showed bone metastases; computed tomography revealed bone spread and abnormal mediastinal lymphadenopathy. Cerebrospinal fluid (CSF) cytology was negative but protein was slightly raised. Palliative hormone therapy was commenced for metastatic breast cancer. She responded quickly with complete resolution of diplopia, ptosis and numbness on her right anterior scalp within 8 weeks of starting anastrozole.

## Discussion

Neoplastic meningitis is diffuse dissemination of malignant cells in the meninges, CSF or both [5]. The most common malignancies are breast and lung cancer, melanoma and B-cell lymphoma [4, 5]. It is diagnosed clinically in 5% of patients but the incidence may be as high as 20% in autopsy series [4]. Diagnosis is by positive CSF cytology, radiologic evidence on neuroimaging, clinical signs and symptoms and abnormal CSF analysis (high white blood cell count, low glucose and elevated protein) [4]. Patients often present with varied neurologic symptoms depending on the area of central nervous system affected [4]. There is a predilection for sites with slow CSF flow and gravity dependent areas allowing tumour cells to seed. Median survival in untreated cases is 4 to 6 weeks [4].

Brain metastases from breast cancer occur usually 42 to 60 months after primary lesion diagnosis [6]. This case is rare because the patient presented with fourth cranial nerve palsy as the first manifestation of undiagnosed metastatic breast malignancy. The history of trauma preceding the onset of double vision was relatively trivial and could not explain the aetiology of the cranial mononeuropathy. Raised inflammatory parameters at the initial presentation were the only clue towards an underlying malignancy. However, the absence of any relevant history in an apparently healthy woman did not alert the physician towards the diagnosis at this point.

Subsequent involvement of other cranial nerves causing ophthalmoplegia pointed to a more sinister condition. By this time she had biopsy proven breast cancer. Metastatic disease was confirmed on an MRI scan with contrast which showed meningeal enhancement. It is surmised that microscopic invasion of the cranial nerve sheaths (not visible on an MRI scan) led to multiple cranial nerve palsies. The diagnosis of metastatic multiple cranial neuropathy so early after diagnosis of primary cancer and as a presenting feature of breast cancer is extremely rare and is hence reported.

## Conclusions

Raised inflammatory parameters in an elderly patient in the absence of biopsy proven GCA or recent infection should alert the ophthalmologist regarding less common, sinister aetiologies. ESR can be raised in metastatic breast cancer [7].

Presentation of metastatic breast cancer with fourth and subsequent third and sixth nerve palsy is extremely rare. This is the first such case to be reported to the best of our knowledge.

## Consent

Written informed consent was obtained from the patient for publication of this case report and accompanying images. A copy of the written consent is available for review by the Editor-in-Chief of this journal.
